# Dynamical Complexity in Geomagnetically Induced Current Activity Indices Using Block Entropy

**DOI:** 10.3390/e27020172

**Published:** 2025-02-06

**Authors:** Adamantia Zoe Boutsi, Constantinos Papadimitriou, Georgios Balasis, Christina Brinou, Emmeleia Zampa, Omiros Giannakis

**Affiliations:** 1 Institute for Astronomy, Astrophysics, Space Applications and Remote Sensing, National Observatory of Athens, Metaxa and Vas. Pavlou St., 15236 Athens, Greece; og@noa.gr; 2 Department of Physics, National and Kapodistrian University of Athens-Panepistimiopolis, 15784 Athens, Greece; sph1900144@uoa.gr; 3 Department of Physics, University of Ioannina, 45110 Ioannina, Greece; ph08777@uoi.gr

**Keywords:** magnetic storms, geomagnetically induced currents, information theory, block entropy, geomagnetic indices, space weather

## Abstract

Geomagnetically Induced Currents (GICs) are a manifestation of space weather events at ground level. GICs have the potential to cause power failures in electric grids. The GIC index is a proxy of the ground geoelectric field derived solely from geomagnetic field data. Information theory can be used to shed light on the dynamics of complex systems, such as the coupled solar wind–magnetosphere–ionosphere–ground system. We performed block entropy analysis of the GIC activity indices at middle-latitude European observatories around the St. Patrick’s Day March 2015 intense magnetic storm and Mother’s Day (or Gannon) May 2024 superintense storm. We found that the GIC index values were generally higher for the May 2024 storm, indicating elevated risk levels. Furthermore, the entropy values of the SYM-H and GIC indices were higher in the time interval before the storms than during the storms, indicating transition from a system with lower organization to one with higher organization. These findings, including the temporal dynamics of the entropy and GIC indices, highlight the potential of this method to reveal pre-storm susceptibility and relaxation processes. This study not only adds to the knowledge of geomagnetic disturbances but also provides valuable practical implications for space weather forecasting and geospatial risk assessment.

## 1. Introduction

Several space weather phenomena are associated with or triggered by magnetic storms or magnetospheric substorms. The impacts of these phenomena range from mild (e.g., interference with aeromagnetic surveys) to severe (e.g., blackouts or collapses of electric power grids). Geomagnetically Induced Currents (GICs) that flow through electrically conductive infrastructure such as power transmission lines are generated by naturally induced geoelectric fields during geomagnetic disturbances such as magnetic storms. GICs can disrupt power grids, potentially causing widespread blackouts and power losses [1,2,3,4]. For instance, the Halloween storms of 2003 resulted in a one-hour power outage in Sweden. Additionally, transformer failures in South Africa during the same event were attributed to prolonged exposure to low-intensity GICs [5,6]. Although GIC intensity is typically greater at high geomagnetic latitudes, where dominant auroral ionospheric currents cause significant magnetic field fluctuations on the ground (especially during disturbed periods, e.g., [7,8]), there has been growing scientific interest and numerous studies over the past two decades in assessing GIC risks in countries located at low and middle latitudes (e.g., [9,10]). These risks strongly depend on factors such as ground conductivity and the topology of the power grid.

Taken together, the multiplicity of recently developed approaches in the field of nonlinear time series analysis offers great potential for uncovering relevant yet complex processes interlinking different geospace subsystems, variables, and spatiotemporal scale; for a review, see [11]. Furthermore, information theory has been shown to be very useful for studies of magnetosphere–ionosphere coupling [12]. In particular, entropy measures such as block entropy have been applied to study the complex dynamic character of the topside ionosphere using satellite measurements from the Swarm mission of the European Space Agency (ESA) [13,14].

Precise computation of GICs in power grids during magnetic storms is a complex two-step process requiring detailed knowledge of transmission line design and the electrical conductivity of the local terrestrial solid crust. While such analysis is challenging, preliminary estimation of the geoelectric field from geomagnetic data is comparatively simple and can be performed in either the time or frequency domain ([15] and references therein). Proxies for GICs such as $dB_{H}/dt$ (e.g., [16,17]) or geomagnetic indices A and K (e.g., [18,19]) have been proposed, but each has limitations. The GIC index has gained attention in low and middle latitude studies (e.g., [10,20,21]) due to its consistency, minimal input requirements, and adaptability for use with ground conductivity models and various infrastructure. Originally introduced by Marshall et al. (2010) [15], the index is derived solely from geomagnetic field data, eliminating the need for information on ground conductivity, ionospheric current system geometry, or infrastructure details. At a single location, the GIC index effectively measures relative risk over time, assuming that the GIC flow at a node (in the pipeline or power transmission network) is proportional to the local geoelectric field. Due to conductivity variations, however, comparisons across regions with different geology reflect the “geoeffectiveness” of the driving magnetic field fluctuations (e.g., [22]) rather than the direct GIC impact.

The magnetic storm of 17 March 2015 (aka the St. Patrick’s Day storm) was the most intense space weather event during solar cycle 24 (2008–2019), when Dst reached -223 nT, known for being relatively weak in terms of solar activity compared to other cycles, including the present (2019–to date). It was triggered by a powerful coronal mass ejection (CME), caused spectacular auroral displays visible as far south as the central United States, and resulted in minor disruptions to satellite operations and radio communications (e.g., [23,24,25]). In contrast, the magnetic storm of 11 May 2024 (aka the Mother’s Day or Gannon storm) has thus far been the most intense of the ongoing solar cycle 25 (Dst reached -412 nT) and the strongest storm over a period of more than 20 years (since the Halloween 2003 superstorm, when Dst reached -422 nT on 20 November 2003). This cycle is significantly more active than its predecessor, with higher sunspot counts and frequent solar eruptions. The 11 May 2024 storm was associated with multiple CMEs that arrived in quick succession, creating a prolonged period of heightened geomagnetic activity. The storm’s effects were even more striking than those of March 2015, with auroras visible at much lower latitudes than usual, including parts of southern Europe and the southern United States (e.g., [26,27]). As previously mentioned, studying GICs at middle latitudes is crucial, especially given the increasing activity of solar cycle 25. The growing frequency and intensity of geomagnetic storms raise concerns about their potential impact on power grids, communication systems, and other critical infrastructure in regions previously considered less vulnerable. A comprehensive and detailed analysis of the Mother’s Day storm and its implications has been provided by Spogli et al. (2024) [28].

Here, we analyze the GIC activity indices using the block entropy around the storm events of March 2015 and May 2024, aiming to highlight possible distinct differences in the degree of order/organization of the coupled solar wind–magnetosphere–ionosphere–ground system as a storm approaches. The latter may help to improve the mitigation of space weather hazards. Section 2 describes the data used in this study and discusses the information theory approaches applied to analyze these data. The rest of the paper deals with the obtained results (Section 3) and their discussion (Section 4).

## 2. Materials and Methods

In this study, we analyze GIC indices along with the standard SYM-H index from March 2015 and May 2024. The SYM-H index represents the longitudinal SYM-(metric) H-(orizontal) component disturbances of the Earth’s magnetic field [29], and is similar to the hourly disturbance storm-time (Dst) index, although it is computed from more ground-based stations and with a finer time resolution of one minute. The Dst (and SYM-H) variation is derived to provide a quantitative measure of geomagnetic disturbances that can be correlated with other solar and geophysical parameters (for the SYM-H index, please visit: https://wdc.kugi.kyoto-u.ac.jp, accessed on 8 November 2024, and NASA’s OmniWeb service https://omniweb.gsfc.nasa.gov [30], accessed on 8 November 2024).

The most intense period of solar cycle 24 in terms of magnetic storm activity was the year 2015, during which the strongest storm of this solar cycle, i.e., the St. Patrick’s Day storm, occurred. A discussion of space weather effects on the ground related to the St. Patrick’s Day storm is provided in Tozzi et al. [10] and Boutsi et al. [21]. Several authors have examined the same storm event using Swarm time series and applying information theory approaches (e.g., [13,31]). The magnetic storm of May 2024, often referred to as the Mother’s Day storm or Gannon storm, was one of the most intense solar events in recent history, providing a critical case study for analyzing the effects of extreme solar activity on Earth’s environment and technological systems. Table 1 shows the strongest magnetic storms of 2015 and 2024 based on minimum SYM-H index values.

Here, we employ data from three magnetic observatories located in France (Chambon la Forêt—CLF), Italy (Castello Tesino—CTS), and Spain (Ebro—EBR). All three data sources come from observatories that are specialized facilities dedicated to recording high-quality measurements of variations in the Earth’s magnetic field. The data are calibrated through manual absolute measurements routinely performed by each observatory’s staff. Table 2 shows the geographic and altitude-adjusted corrected geomagnetic (AACGM) coordinates of each observatory estimated for the epoch 2015.0, along with the corresponding L-shell values.

### 2.1. GIC Index

The GIC index is calculated using the method outlined in Marshall et al. (2010) [15], which applies a frequency domain filter to the geographic North (X) or East (Y) component of the geomagnetic field’s horizontal intensity. This filter function represents the “surface impedance” for a half-space uniformly-conducting one-dimensional (1D) Earth model. It is based on the magnetotelluric principle, which relates the orthogonal electric and magnetic field components through a single-valued complex transfer function (e.g., [32]). The GIC index for either the X or Y direction is obtained by taking the absolute value of the inverse transformation back into the time domain. As described by Marshall et al. (2010) [15], users can choose the index (GICx or GICy) that best aligns with their specific infrastructure system.

Magnetometer datasets sampled at a 1-min cadence were carefully inspected for spikes (outliers) and small gaps. The analysis revealed only a few instances of anomalous spikes, which were likely caused by instrumental noise or transient disturbances. To ensure data quality and continuity, these outliers were addressed through minor preprocessing techniques (i.e., linear interpolation was applied to smooth the affected data points). As pointed out by Tozzi et al. (2019b) [20], linear interpolation does not play a significant role in the results, and if it does affect the GIC index the only effect will be to underestimate it. Next, all time series were detrended in order to remove the linear trend from the geomagnetic field data. According to Marshall et al. (2011) [33], the formulas applied to the detrended geomagnetic field data using a 1-day moving window [10] are as follows:

(1) $$\begin{array}{cc} GICx(t) & {=|FFT\left\{Y\left(f\right)Z\left(f\right)\right\}}^{-1}| \end{array}$$

(2) $$\begin{array}{cc} GICy(t) & {=|FFT\left\{X\left(f\right)Z\left(f\right)\right\}}^{-1}| \end{array}$$

(3) $$\begin{array}{cc} Z(f) & =e^{i\frac{\pi }{4}}\sqrt{\frac{f}{f_{N}}} \end{array}$$

where X(f) and Y(f) are the North and East components of the magnetic field in the frequency domain, $FFT{\left\{\ldots \right\}}^{-1}$ is the inverse Fourier transform of the formula inside the brackets, || represents the absolute value, Z(f) is the filter function with normalized amplitude and phase characteristics, *f* is the frequency, and $f_{N}$ is the Nyquist frequency ($f_{N}=8.3$ mHz for a sampling rate of 1 value/min). All calculations were performed on monthly time series.

### 2.2. Block Entropy

In his seminal paper in the late 40 s, Claude Shannon used the famous Boltzmann’s H theorem to quantify the amount of information transmitted by a telecommunication signal [34]. Assuming that a system has *M* distinct states, with $p_{i}$ as the probability of occurrence of state *i*, then the information carried by a signal broadcast by the system can be computed by calculating its entropy:

(4) $$H=-\sum_{i}p_{i}log\left(p_{i}\right).$$

Zero entropy means that the system remains in only one of its states, meaning that its behavior has collapsed to a single monotonous condition; on the other hand, maximum entropy (log(M)) means that the system exhibits fully chaotic random behavior. Of course, in real-world applications it is practically impossible to discern the state in which a system really is. However, it is easy to consider the values of one of its observable parameters and digitize them; thus, when a parameter lies within a certain range of values $X_{k}$ to $X_{k+1}$, the system can be considered to be in a particular state *k*. By computing the probability of occurrence of each of these states, the Shannon entropy can be calculated.

In this simplified approach, the temporal information of these states is ignored and only their statistical distribution is taken into consideration. A better method is to digitize the series and then examine it in terms of “blocks” of consecutive time instants in order to assess what types of patterns emerge. As an example, the signal of a binary system that exhibits only two states, e.g., states 0 and 1, can be digitized in a binary sequence, which can then be parsed by blocks of a certain length, e.g., 3. The new “states” of this signal are now all possible combinations of these original two states; for example, in the 3-length blocks, the new states are 000, 001, 011, and so on. The probabilities of these new states are calculated and the entropy of the block length is estimated, e.g., H(3), which typically yields a much better estimation of the information content of the signal [35,36].

In this work, we consider as signals the time series of the GIC index of the selected observatories (both *X* and *Y* components), which is read in “windows” of 5000 points. For each window, the signal is digitized in a binary sequence, using the window mean as the threshold, which produced the best results among a variety of other thresholds we examined. Then, the entropy for blocks of various lengths is calculated. As the block length ‘*n*’ increases, the number of possible blocks also increases, scaling as $2^{n}$ for binary sequences (or in general, $a^{n}$, with ‘*a*’ being the number of symbols). However, this means that in order to have adequate statistics, a larger window size will be needed. If we assume that we require approximately 100 occurrences of each block for accurate statistics, then in the case of full randomness, where all blocks appear with equal probability, a window of 5000 points is enough for 5000/100=50 different blocks. This corresponds to a block size of approximately 6 (because $2^{6}=64$); thus, the results for the block entropy H(6) are presented (although the same conclusions can be drawn for other similar block lengths). The window then moves by 50 points forward and the process is repeated. In this way, the changes in the dynamical nature of the system can be detected. As a system transitions from its random background noise state to a more particular behavior, e.g., when a magnetic storm is occurring, its entropy decreases, and this drop in entropy will be evident in the analysis of the corresponding windows.

## 3. Results

Magnetosphere–ionosphere coupling involves intricate interactions and feedback processes that are challenging to analyze using traditional methods. To address this, we employ block entropy analysis, a method rooted in symbolic dynamics techniques, to examine time series data from the SYM-H index as well as the GICx and GICy indices.

Our analysis focuses on data collected from three mid-latitude observatories (CLF, CTS, and EBR) during the strongest magnetic storms of the previous and current solar cycles. By applying block entropy, we aim to capture the underlying patterns and information transfer within these indices, shedding light on the temporal evolution and complexity of geomagnetic activity. This approach not only enhances our understanding of magnetosphere–ionosphere interactions but also provides a novel perspective for assessing the ground effects of space weather events.

**Figure 1:**
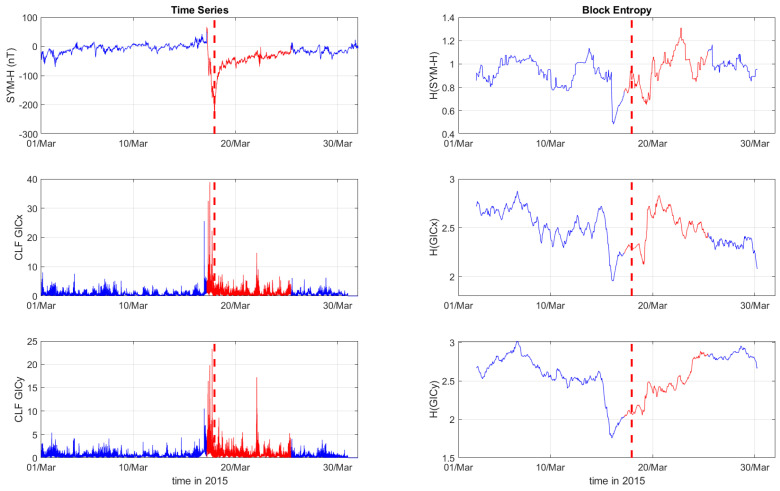
Time series of SYM−H, GICx, and GICy of the Chambon la Forêt observatory (**left**) and corresponding block entropies (**right**) for March of 2015. The red color marks the duration of the storm, while the dashed red line corresponds to the peak of the storm.

**Figure 2:**
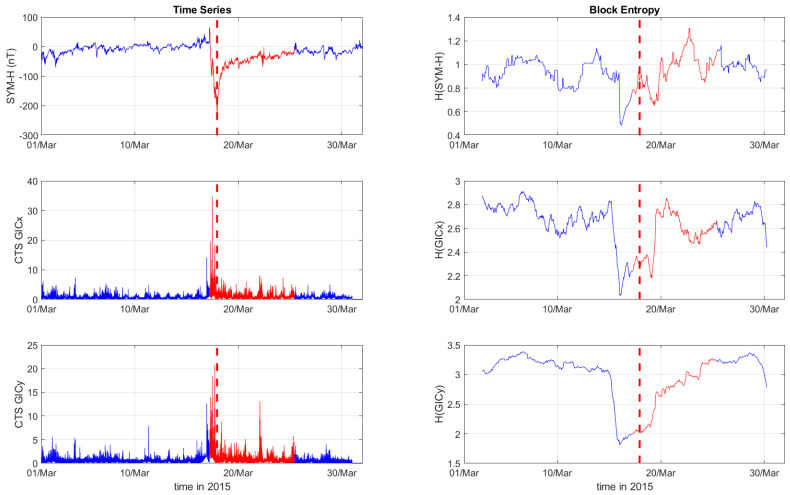
Time series of SYM−H, GICx, and GICy of the Castello Tesino observatory (**left**) and corresponding block entropies (**right**) for March of 2015. The red color marks the duration of the storm, while the dashed red line corresponds to the peak of the storm.

**Figure 3:**
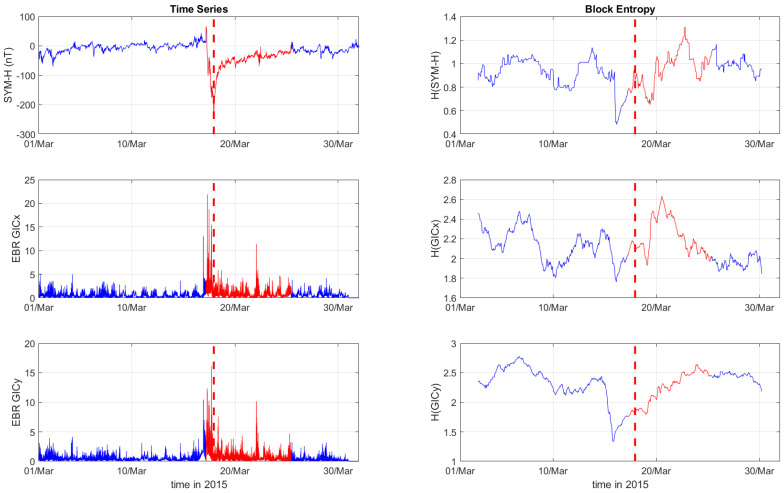
Time series of SYM−H, GICx, and GICy of the Ebro observatory (**left**) and corresponding block entropies (**right**) for March of 2015. The red color marks the duration of the storm, while the dashed red line corresponds to the peak of the storm.

**Figure 4:**
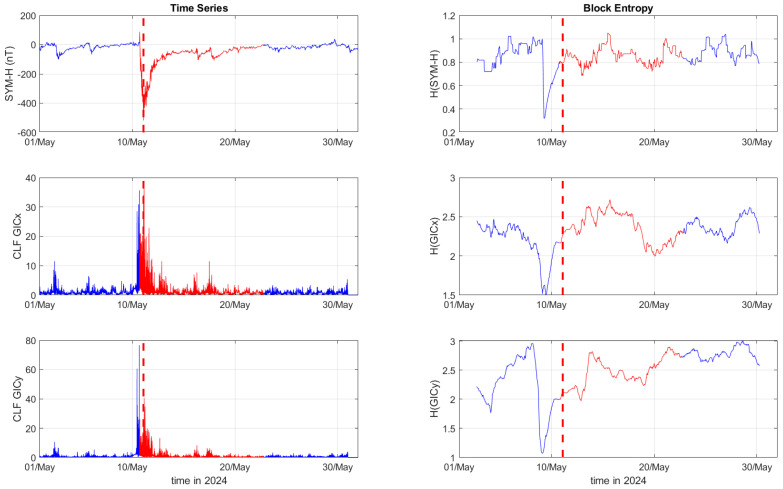
Time series of SYM−H, GICx, and GICy of the Chambon la Forêt observatory (**left**) and corresponding block entropies (**right**) for May of 2024. The red color marks the duration of the storm, while the dashed red line corresponds to the peak of the storm.

**Figure 5:**
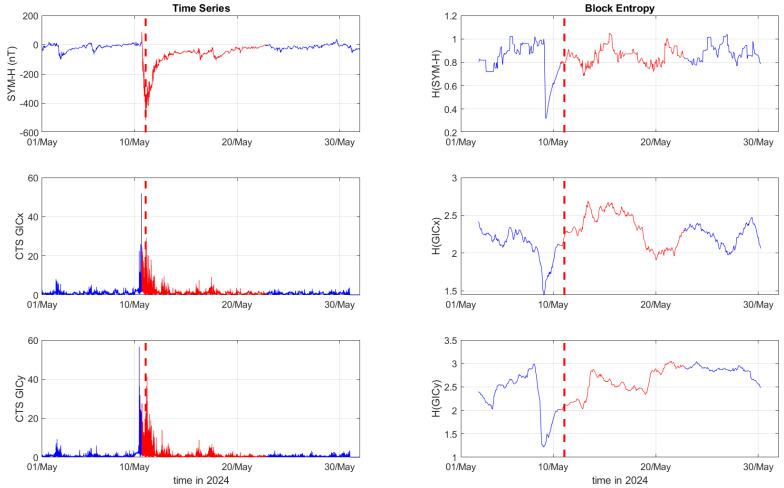
Time series of SYM−H, GICx, and GICy of the Castello Tesino observatory (**left**) and corresponding block entropies (**right**) for May of 2024. The red color marks the duration of the storm, while the dashed red line corresponds to the peak of the storm.

**Figure 6:**
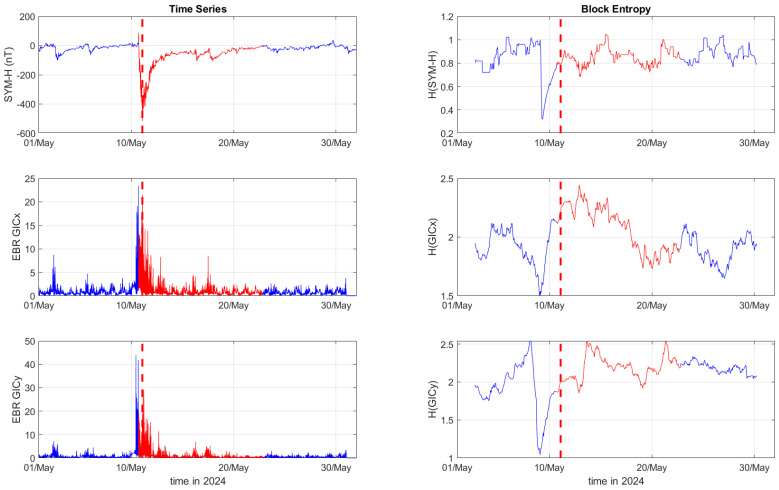
Time series of SYM−H, GICx, and GICy of the Ebro observatory (**left**) and corresponding block entropies (**right**) for May of 2024. The red color marks the duration of the storm, while the dashed red line corresponds to the peak of the storm.

Figure 1, Figure 2 and Figure 3 depict the temporal variation of the SYM-H, GICx, and GICy indices (left columns) for CLF, CTS, and EBR, respectively, along with their corresponding block entropies (right columns) for March 2015. Similarly, Figure 4, Figure 5 and Figure 6 show the temporal variation of the SYM-H, GICx, and GICy indices (left columns) for CLF, CTS, and EBR, respectively, along with their corresponding block entropies (right columns) during May 2024.

During the St. Patrick’s Day storm in 2015 (Figure 1, Figure 2 and Figure 3), the SYM-H index reached a minimum of −234 nT at 22:47:00 UT. The GICy index reached the peak values of 23.3 for CLF, 20.7 for CTS, and 16.2 for EBR. Similarly, the GICx index obtained the maximum values 39.0 for CLF, 34.7 for CTS, and 21.9 for EBR. During the Mother’s Day (or Gannon) storm in 2024 (Figure 4, Figure 5 and Figure 6), the SYM-H index dropped to −518 nT at 02:14:00 UT on May 11. The GICy index reached the maximum values of 76.6 for CLF, 56.6 for CTS, and 44.0 for EBR. Similarly, the GICx index obtained the peak values 38.5 for CLF, 51.9 for CTS, and 23.4 for EBR. These observations are shown in the left panels of Figure 1, Figure 2, Figure 3, Figure 4, Figure 5 and Figure 6.

The right panels of Figure 1, Figure 2 and Figure 3 display the block entropies for the SYM-H, GICx, and GICy indices during March 2015. These panels highlight a characteristic drop in entropy values at 4:10 UT on March 16 preceding the intense magnetic storm on 17 March 2015, signaling the storm’s arrival before the SYM-H index reached its minimum value. This pattern is evident in the SYM-H, GICx, and GICy indices across all three locations (i.e., CLF, CTS, and EBR), with more pronounced manifestation for CLF and CTS. Similarly, the right panels of Figure 4, Figure 5 and Figure 6 illustrate the block entropies for the SYM-H, GICx, and GICy indices during May 2024. The intense magnetic storm on 11 May 2024 is marked by a distinct drop in entropy values almost two days before the storm at 7:10 UT on May 9. In this case, the SYM-H index exhibits a sharper and more abrupt decline in entropy closer to the storm’s onset, with a faster recovery compared to the March 2015 storm. A similar trend is followed by the block entropies of the GIC indices for CLF, CTS, and EBR; however, the drop in entropy values lasts longer for GICy and recovers more slowly.

## 4. Discussion

GICs at low and middle latitudes are associated with ionospheric source fields distinct from those observed at high latitudes, such as auroral electrojets. Kappenman (2005) [37] identifies the source of sustained GICs at low and middle latitudes as high rates of geomagnetic field variation, typically driven by impulsive increases in solar wind dynamic pressure or ring current intensification. In these regions, the maximum time variation of the horizontal magnetic field component ($dB_{H}/dt$) often occurs during the abrupt storm onset rather than the main phase, resulting in heightened vulnerability during these periods [38]. The majority of intense GICs appear to result from sudden impulses (SIs) or sudden storm commencements (SSCs) that typically precede magnetic storms. SIs are triggered by enhanced magnetopause currents caused by the compression of the magnetosphere by solar plasma arriving after extreme solar events such as coronal mass ejections (CMEs) or co-rotating interaction regions (CIRs) [39]. Notably, large voltages at middle latitudes during the recovery phase of magnetic storms are linked to Pc5 pulsations (e.g., [20,40]).

Marshall et al. (2011) [33] introduced a GIC risk level scale based on GIC index thresholds derived from a relative probability model. The risk levels (very low, low, moderate, high and extreme) correspond to GICy index values of ≤50, 50–100, 100–250, 250–600, and >600, respectively. For GICx, the thresholds are halved (≤25, 25–50, 50–125, 125–300, and >300). Tozzi et al. (2019a) [10] have suggested calibrating these thresholds for middle-latitude countries, as the model used by Marshall et al. (2011) [33] was developed based on historical GIC activity recorded in both middle- and high-latitude regions, including Canada, China, Japan, New Zealand, Scotland, Sweden, the UK, and the USA. Because GIC occurrence and intensity are strongly influenced by latitude-dependent geomagnetic conditions, applying uncalibrated thresholds to middle-latitude regions may lead to an underestimation of risk. Thresholds derived from high-latitude data may not fully capture the distinct geomagnetic and geoelectric environments of mid-latitude power grids, potentially overlooking moderate but still hazardous storm conditions that could impact infrastructure. However, calibrating these thresholds requires extensive regional data analysis, which is a task beyond the scope of this study; thus, we have used the original thresholds to provide an initial risk level assessment while acknowledging their limitations and potential for underestimating actual GIC exposure.

Table 3 presents the maximum GIC index values for the two storm events. During the St. Patrick’s Day storm, most GIC index values are in the “very low” risk level on the GIC risk scale [33], except for the GICx index at CLF and CTS, which exceed this level. For the Mother’s Day (or Gannon) storm, most maximum GIC index values are within the “low” risk level, except for the GICx index at CTS, which reaches the “moderate” level, and the GICx and GICy indices at EBR, which fall below the “low” range.

We observe that the GIC index values are generally higher for the May 2024 storm, except for the GICx index at CLF, where the maximum value is slightly greater during the St. Patrick’s Day storm (39.0) compared to the Mother’s Day (or Gannon) storm (38.5). Additionally, GIC index values tend to decrease from north to south (CLF to EBR), with the sole exception of GICx at CTS, which reaches a peak value of 51.9. This deviation at CTS could be attributed to various factors, such as ionospheric current systems (e.g., storm-time ring currents), which might have had stronger or more focused activity near CTS during this event, amplifying the observed GIC response. Variations in the orientation and intensity of the driving geomagnetic field fluctuations at CTS could also play a role. Further analysis, including detailed modeling of local conductivity profiles and ionospheric current configurations, would be necessary in order to fully understand this exception.

GICs are directly linked to geoelectric fields; however, their estimation remains complex and time consuming in computational needs. Accurately determining GICs requires detailed knowledge of ionospheric currents/geomagnetic disturbances as well as the three-dimensional (3D) distribution of the Earth’s electrical conductivity [41]. The GIC index provides a simple yet effective method for assessing relative risk over time, particularly at middle and low latitudes (similar to how dB/dt is often used in high-level studies); however, the GIC index appears to offer certain advantages in this context, and can serve as a useful indicator of relative risk even without detailed ground conductivity information. Incorporating knowledge of the Earth’s conductivity structure could enhance GIC estimations and help to explain variations in GIC values across different observatories. For example, it is well established that the horizontal geoelectric field is amplified in coastal regions due to the significant lateral conductivity contrast at the ocean–land interface, i.e., the coast effect distortion [42]. Understanding such regional effects is crucial for improving GIC predictions and assessing their potential impact more accurately.

We find that the entropy values of the SYM-H and GIC indices are higher in the time interval before the storm than during its main and recovery phases. Therefore, our findings clearly indicate a transition from a state with higher complexity or less order prior to the storm to a state with less complexity or increased order during the storm and its recovery phase. These findings align with earlier studies analyzing the entropy of geomagnetic activity indices [43] as well as more recent studies on the entropy analysis of Swarm magnetic and electric field data [13,31] and Swarm indices [44].

More specifically, for the time period before the onset of each storm, all entropy values for the 2024 case are lower than their corresponding values for the 2015 storm, which is apparent from the plots and is also verified by comparing the statistical distributions of the pre-storm values and by paired *t*-tests performed to ensure that the differences were significant and could not be attributed to random variations. This is an interesting finding, and might indicates that the entire magnetosphere–ionosphere coupling system was already disturbed before the storm’s onset on the 10th of May, i.e., that the system was already in a preconditioned state, which could explain the pronounced effect of the interplanetary disturbance (for a previous example of a similar case, see [45]), leading to the drop in Dst/SYM-H indices indicating the greatest magnetic storm of the current solar cycle. However, further analyses would be required to support this hypothesis.

Another feature of note for the 2015 storm is that the relative drop in block entropy for the SYM-H index is about 50%, reaching values of approximately 0.5 compared to its pre-storm level of around 1. For the GIC-based indices the reduction is smaller percentage-wise but appears wider with respect to time, despite the fact that the same temporal window was used for the analysis of all signals. For the 2024 case, the relative reduction of entropy for the GIC-based indices is greater than that of the SYM-H index, showing that this storm was not only a more intense event in space, but that it had a greater geoeffect on the ground as well. For both storms, the temporal profiles of the drop in entropy are wider than in the SYM-H index. This latter observation could be a feature of the methodology responsible for the production of the GIC index itself, but could also indicate that the block entropy can be utilized as a more descriptive measure of the temporal extent of geomagnetic disturbances.

Finally, the return to pre-storm levels of the block entropy in the GIC index, especially its Y component, is a much more gradual process compared to the return in the SYM-H index itself or in its corresponding block entropy. This highlights how the entropy of GICy provides a better viewpoint for studying the disturbance of the geomagnetic environment, as it clearly shows that the effect of magnetic storms can extend to several weeks after the main phase while the entire system slowly relaxes back to its previous state, even after the SYM-H index itself has returned to typical quiescence values.

## 5. Conclusions

Information theory offers a robust framework for quantifying the information content and dynamics of complex systems, including the magnetosphere–ionosphere coupling system; for more, see the recent reviews by Balasis et al., 2023 [11] and McGranaghan, 2024 [46].

In particular, information theory has been successfully applied to study the magnetosphere of the Earth [47,48,49,50,51,52,53,54] and the Sun [55,56,57,58,59]. Information theory can help to untangle the solar wind drivers of magnetospheric phenomena through constructs such as conditional mutual information [51,52,60,61,62].

In this study, we have analyzed 1-month time series of the SYM-H geomagnetic activity index and GIC indices for March 2015 and May 2024 from three middle-latitude observatories using the information-theoretical measure of block entropy.

By producing the GIC indices from ground-based data, it is possible to see the enhanced effect of the Mother’s Day (or Gannon) superstorm of 2024 compared to the St. Patrick’s event of 2015, as half of the indices rise to the next risk level (from “very low” to “low” and from “low” to “moderate”). Additionally, the first increase of the GIC index takes place before the storm onset (especially for the Y component), while the peak of the index also occurs before the peak of the storm, showcasing how GIC indices can be a valuable tool for the study of geomagnetic disturbances related to both the ring current and the various ionospheric current systems.

Together with the above analysis, our block entropy analysis illustrates that the geomagnetic system exhibited lower entropy values before the onset of the May 2024 storm compared to the March 2015 case, indicating that the relevant current systems might have already been disturbed or not completely returned to their quiet phase, making them more susceptible to new disturbances. Furthermore, our entropy analysis of the GIC indices shows wider temporal profiles and provides a new viewpoint describing the relaxation process of the system as it gradually returns to its pre-storm phase. This kind of analysis can provide new perspectives to scientists and space operators, helping them to better estimate the effectiveness of incoming solar events and the general susceptibility of geospace to such disruptions.

The present study stands out for its relevance and timeliness, as the Sun has reached its solar maximum period at the end of 2024, which could continue into 2025. It addresses critical aspects of space weather and its implications for technological systems on the ground, particularly in the middle latitudes. The innovative methodology and related findings in this paper provide valuable contributions to understanding magnetosphere–ionosphere coupling and mitigating space weather risks. As the solar cycle evolves, we will aim to extend this work by including similar analysis of data from additional magnetic observatories as well as data on more storm events, including longer time windows. Along with data from low-Earth orbit (LEO) satellites such as the ESA’s Swarm mission, this could provide the ability to capture longer-term patterns and recovery dynamics.

## Figures and Tables

**Table 1:** Strongest magnetic storms of 2015 (solar cycle 24) and 2024 (solar cycle 25). The storm date, time, and minimum SYM-H index value reached are provided in the second, third, and fourth columns, respectively.

Case	Storm Date	Storm Time (UT)	SYM-H (nT)
#1	17 March 2015	22:47:00	−234
#2	11 May 2024	02:14:00	−518

**Table 2:** Geographic and altitude-adjusted corrected geomagnetic (AACGM) coordinates of the magnetic observatories in the present study; L-shell values (in geocentric coordinates, height above sphere Re = 6371.2 km) are also shown in the last column.

Observatory	GLat (°N)	GLon (°E)	Alt. (m)	MLat (°N)	MLon (°E)	L (Re)
Chambon la Forêt (CLF)	48.025	2.260	145	42.801	78.884	1.909
Castello Tesino (CTS)	46.047	11.649	1175	40.404	86.434	1.758
Ebro (EBR)	40.957	0.333	531.5	33.399	75.867	1.472

**Table 3:** Maximum GIC index values calculated for each magnetic storm and their corresponding risk levels according to Marshall et al. (2011) [33].

	March 2015		May 2024	
**Observatory**	GICy	GICx	GICy	GICx
	*Risk Level*	*Risk Level*	*Risk Level*	*Risk Level*
CLF	23.3	39.0	76.6	38.5
	*Very Low*	*Low*	*Low*	*Low*
CTS	20.7	34.7	56.6	51.9
	*Very Low*	*Low*	*Low*	*Moderate*
EBR	16.2	21.9	44.0	23.4
	*Very Low*	*Very Low*	*Very Low*	*Very Low*

## Data Availability

The results presented rely on data provided by NASA’s OmniWeb service https://omniweb.gsfc.nasa.gov, accessed on 8 November 2024. Ground magnetometer data from CLF (France) and EBR (Spain) are provided by INTERMAGNET www.intermagnet.org, accessed on 8 November 2024. The CTS magnetic observatory is operated by INGV, Rome. Data used in this paper are available at http://www.wdc.bgs.ac.uk/dataportal/, accessed on 8 November 2024 and http://geomag.rm.ingv.it/index.php, accessed on 8 November 2024.
